# Supplementary material for: A Combination of Heavy Metals and Intracellular Pathway Modulators Induces Alzheimer Disease-like Pathologies in Organotypic Brain Slices
Source: Biomolecules. 2024 Jan 30;14(2):165. doi: 10.3390/biom14020165 (PMC10887098; doi:10.3390/biom14020165)
Supplement: Supplementary file 1 [file biomolecules-14-00165-s001.zip › biomolecules-2785432-supplementary.pdf]

Supplementary Figure 1- Additional data supporting the main results

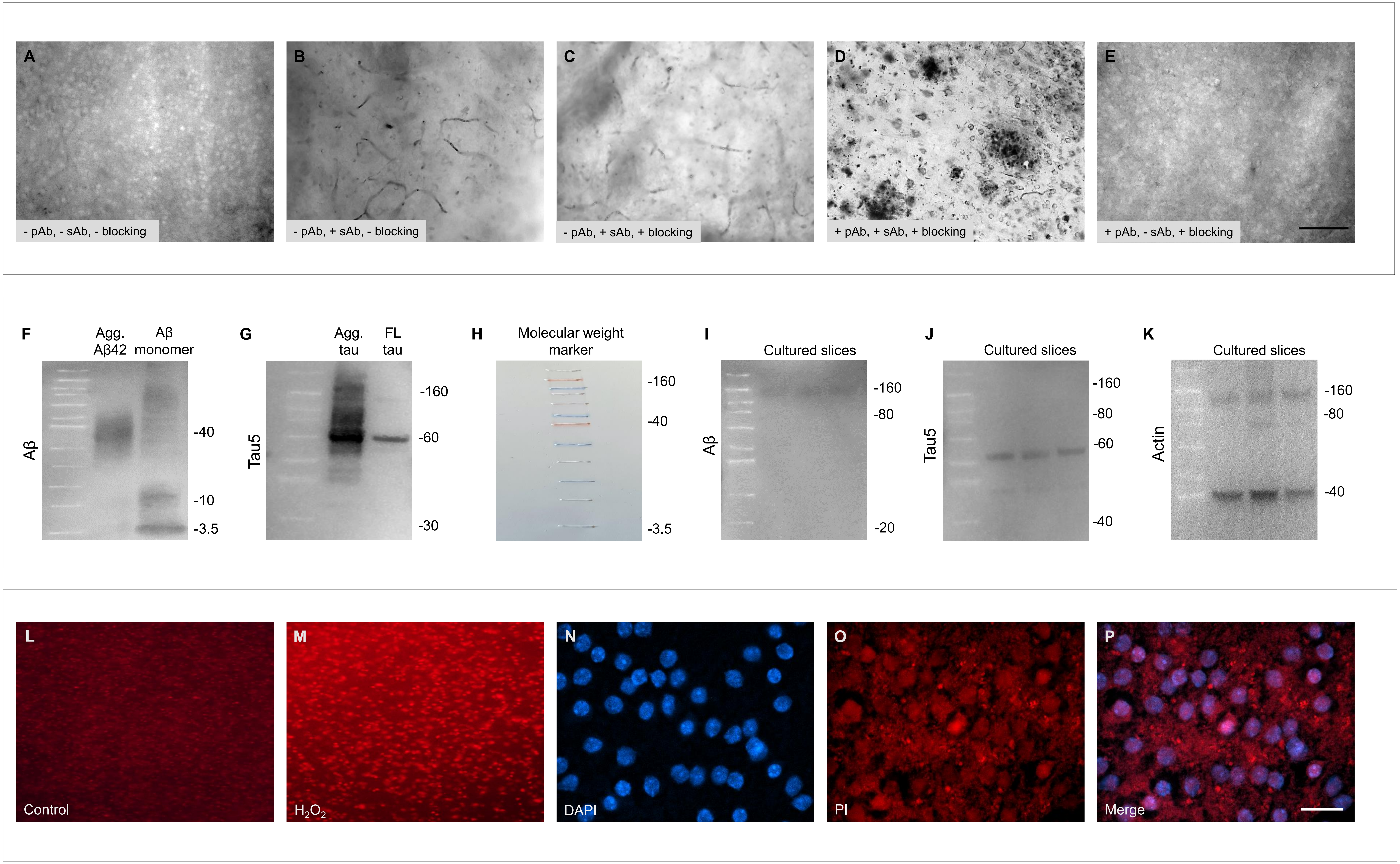

Supplementary Figure 1.
Slices from adult transgenic mice overexpressing the amyloid precursor protein with the Swedish, Dutch, Iowa mutations (APP_SDI, 6-9 months old) were generated and immediately fixed with 4% paraformaldehyde (PFA). The post-fixed slices were subject to the immunohistochemistry protocol. Panels **(A-D)** show representative images of post-fixed adult slices treated: **(A)** without primary antibody (pAb), secondary antibody (sAb) and blocking step with the mouse-on-mouse (M.O.M.) blocking step. **(B)** without pAb and blocking step but with the anti-mouse sAb. **(C)** without pAb but with the anti-mouse sAb and blocking step. **(D)** with amyloid-beta (Aβ) pAb, sAb and the blocking step. **(E)** with Aβ pAb, blocking step but without anti-mouse sAb. Only panel **(D)** shows specific staining for Aβ plaques while the other images reveal background staining. Scale bar in **(E)** = 100 µm in **(A-E)**.
Panels **(F-K)** display Western blots: **(F)** Aβ antibody clone 6E10 detects the aggregated human Aβ42 peptide and the monomeric Aβ form. **(G)** Tau5 antibody detects the aggregated full-length tau as a smear and full-length tau at the expected 60 kDa mark. **(H)** A picture of the PDVF membrane displaying the molecular weight marker that is used on every blot. **(I)** Organotypic brain slices from postnatal mice (day 8-10) were cultured for 8 weeks. Subsequently, the slices were pooled together, sonicated and the supernatant was used to probe for Aβ through a Western blot. Aβ antibody clone 6E10 detects a faint signal at the 160 kDa mark, which may be murine amyloid precursor protein (APP). **(J)** Tau5 detects tau in cultured slices at the expected 60 kDa mark. **(K)** Actin signal is also detectable on the blots at the 40 kDa mark.
Panels **(L-P)** show representative images of organotypic brain slices of postnatal mice (day 8-10), cultured for 2 weeks, and stained with 2 µg/ml propidium iodide (PI) for 30 minutes prior to fixation with 4% PFA. **(L)** A control slice shows background PI staining. **(M)** 2 µl of hydrogen peroxide per 1 ml of media was added to the slice media 2 days before PI staining and fixation acting as the positive control. This image reveals considerable cell death as detected by the bright fluorescent signal from PI. **(N)** DAPI detects nuclei from the fixed slices. **(O)** PI staining of a dead cell. **(P)** The merged image of panels **(N-O)** show a colocalization of both signals. Scale bar in **(P)** = 250 µm in **(L-M)**, 20 µm in **(N-P)**.

Supplementary Figure 2- AT8+ and Aβ+ immunoreactivity in ventral regions of postnatal wild-type slices upon treatment with various pharmacological agents

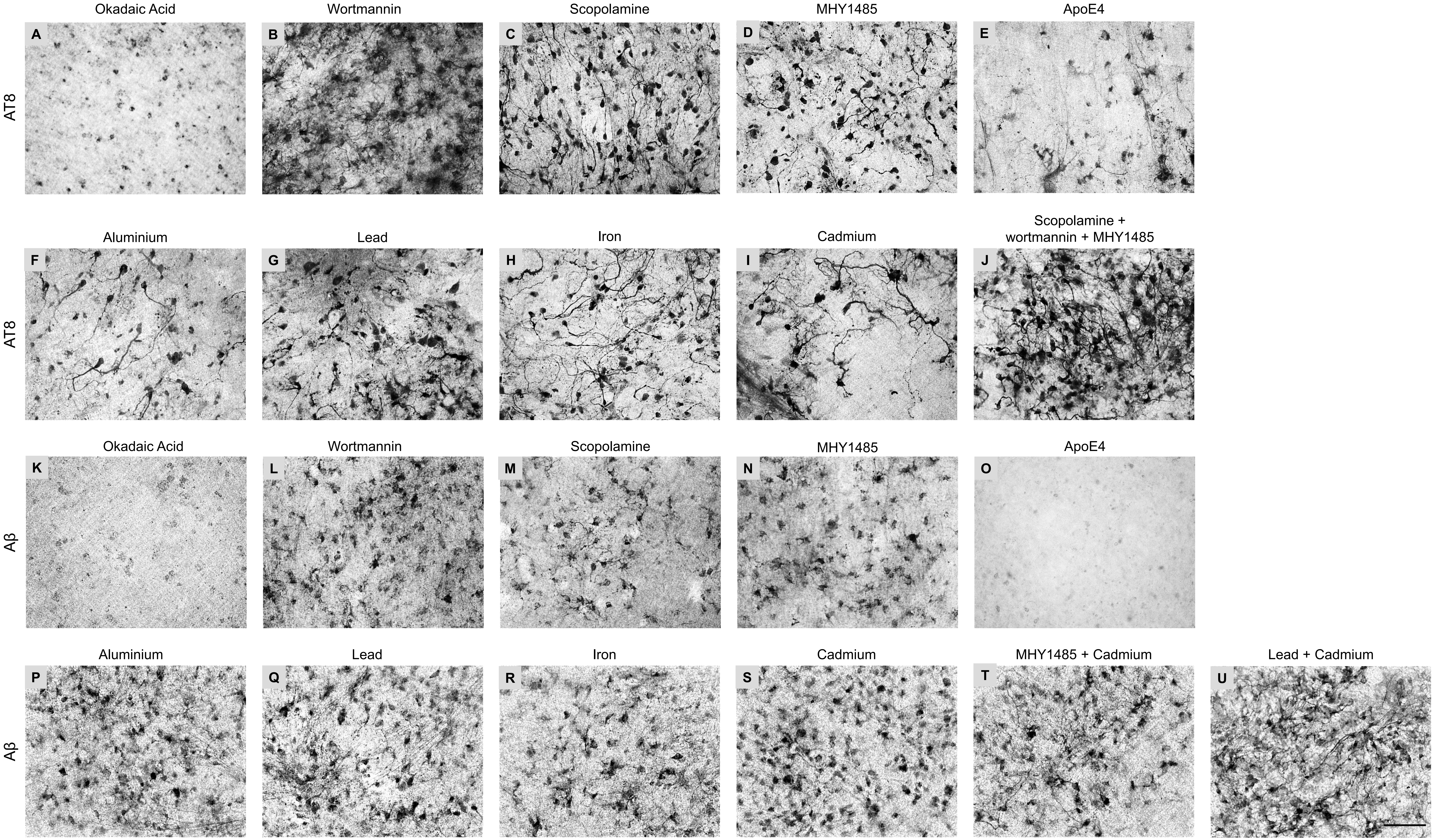

Supplementary Figure 2.

Slices from postnatal wild-type (WT) mice (day 8-10) were generated and collagen hydrogels containing both human amyloid-beta 42 (hAβ42) and P301S aggregated tau (aggTau) were applied after 1 week. After 4 weeks, slice media was supplemented with various pharmacological agents for an additional 4 weeks for a total of 9 week culture period. Slices were fixed in 4% PFA and immunohistochemistry was performed with Aβ antibody clone 6E10 and AT8 tau antibody.

Panels **(A-J)** display representative images of AT8+ immunoreactivity of slices treated with: **(A)** Okadaic acid (100 nM) **(B)** Wortmannin (10 nM) **(C)** Scopolamine (50 nM) **(D)** MHY1485 (50 nM) **(E)** ApoE4 (10 ng/ml) **(F)** Aluminium chloride (100 nM) **(G)** lead acetate (100 nM) **(H)** Iron sulfate (100 nM) **(I)** Cadmium chloride (100 nM) **(J)** Scopolamine+ wortmannin+ MHY1485 (50 nM, 10 nM, 50 nM).

Panels **(K-U)** display representative images of Aβ+ immunoreactivity of slices treated with: **(K)** Okadaic acid (100 nM) **(L)** Wortmannin (10 nM) **(M)** Scopolamine (50 nM) **(N)** MHY1485 (50 nM) **(O)** ApoE4 (10 ng/ml) **(P)** Aluminium chloride (100 nM) **(Q)** lead acetate (100 nM) **(R)** Iron sulfate (100 nM) **(S)** Cadmium chloride (100 nM) **(T)** MHY1485+ cadmium chloride (50 nM, 100 nM) (U) Lead acetate+ cadmium chloride (100 nM each). Scale bar in U = 100 µm in **(A-U).**
